# Automated Image-Based Wound Area Assessment in Outpatient Clinics Using Computer-Aided Methods: A Development and Validation Study

**DOI:** 10.3390/medicina61061099

**Published:** 2025-06-17

**Authors:** Kuan-Chen Li, Ying-Han Lee, Yu-Hsien Lin

**Affiliations:** 1 Division of Plastic Surgery, Department of Surgery, Shin Kong Wu Ho-Su Memorial Hospital, No. 95, Wenchang Road, Shilin District, Taipei 111, Taiwan; kuangcheng99@gmail.com (K.-C.L.); lancelordlin@gmail.com (Y.-H.L.); 2 Department of General Medicine, Shin Kong Wu Ho-Su Memorial Hospital, No. 95, Wenchang Road, Shilin District, Taipei 111, Taiwan

**Keywords:** wound area, K-means clustering, computer aided

## Abstract

*Background and Objectives:* Traditionally, we evaluate the size of a wound by using Opsite Flexigrid transparent film dressing, placing it over the wound, tracing the edges of the wound, and then calculating the area. However, this method is both time-consuming and subjective, often leading to varying results depending on the individual performing the assessment. In this study, our goal is to provide an objective method to calculate the wound size and solve variations in photo-taking distance caused by different medical practitioners or at different times, as these can lead to inaccurate wound size assessments. To evaluate this, we employed K-means clustering and used a QR code as a reference to analyze images of the same wound captured at varying distances, objectively quantifying the areas of 40 wounds. This study aims to develop an objective method for calculating the wound size, addressing variations in photo-taking distance that occur across different medical personnel or time points—factors that can compromise measurement accuracy. By improving consistency and reducing the manual workload, this approach also seeks to enhance the efficiency of healthcare providers. We applied K-means clustering for wound segmentation and used a QR code as a spatial reference. Images of the same wounds taken at varying distances were analyzed, and the wound areas of 40 cases were objectively quantified. *Materials and Methods:* We employed K-means clustering and used a QR code as a reference to analyze wound photos taken by different medical practitioners in the outpatient consulting room. K-means clustering is a machine learning algorithm that segments the wound region by grouping pixels in an image according to their color similarity. It organizes data points into clusters based on shared features. Based on this algorithm, we can use it to identify the wound region and determine its pixel area. We also used a QR code as a reference because of its unique graphical pattern. We used the printed QR code on the patient’s identification sticker as a reference for length. By calculating the ratio of the number of pixels within the square area of the QR code to its actual area, we applied this ratio to the detected wound pixel area, enabling us to calculate the wound’s actual size. The printed patient identification stickers were all uniform in size and format, allowing us to apply this method consistently to every patient. *Results:* The results support the accuracy of our algorithm when tested on a standard one-cent coin. The paired *t*-test comparing the first and second photos shot yielded a *p*-value of 0.370, indicating no significant difference between the two. Similarly, the *t*-test comparing the first and third photos shot produced a *p*-value of 0.179, also showing no significant difference. The comparison between the second and third photos shot resulted in a *p*-value of 0.547, again indicating no significant difference. Since all *p*-values are greater than 0.05, none of the test pairs show statistically significant differences. These findings suggest that the three randomly taken photo shots produce consistent results and can be considered equivalent. *Conclusions:* Our algorithm for wound area assessment is highly reliable, interchangeable, and consistently produces accurate results. This objective and practical method can aid clinical decision-making by tracking wound progression over time.

## 1. Introduction

**Figure 1:**
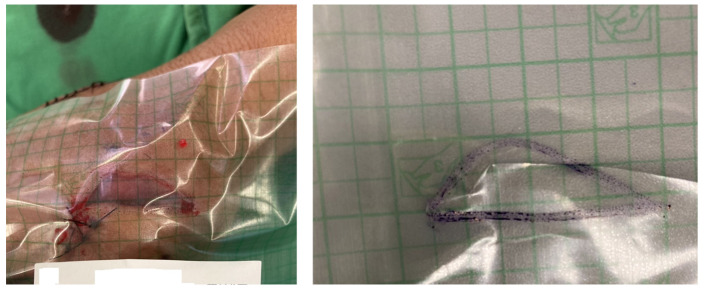
The traditional manual method used for wound area estimation.

Traditionally, in the absence of electronic devices, the most reliable method for wound size assessment is the use of Opsite Flexigrid transparent film dressing [1]. In this method, the Flexigrid film is placed directly over the wound, and a marking pen is used to trace the wound’s edges (Figure 1). By calculating the marked area, clinicians can estimate the wound size. However, most wounds have irregular and uneven margins, making it difficult to accurately measure their size using standard geometric formulas. Furthermore, this manual process is often time-consuming and prone to human error.

Accurate wound size assessment is critical in clinical practice, as it directly affects treatment decisions, healing predictions, and patient outcomes. In contrast, inaccurate measurements can significantly influence surgical management and lead to delayed healing [2,3]. For instance, effective burn management largely depends on accurate wound size estimation using the total body surface area (TBSA). This estimation guides initial fluid resuscitation and nutritional support, both of which are essential for stabilizing the patient and promoting healing. Overestimating or underestimating the wound surface area can result in inadequate fluid administration or fluid overload, which may subsequently affect the prognosis and survival rates [4]. For patients with chronic wounds, the changes in wound size, whether becoming larger or smaller, will give clinicians a clue of the treatment plan. Today, we have various treatment modalities available for chronic wounds [5]. There are now several methods available for assessing the wound area, including digital photography [6,7,8,9,10], 3D wound modeling, optical imaging, high-frequency ultrasound, fluorescence imaging, and artificial intelligence [11,12]. In our approach, we use commonly available smartphones (such as iPhone, Samsung, and Asus) in the outpatient clinic to capture wound images. These images are then processed using AI-assisted automated segmentation to outline the wound area directly from the photo.

To accurately measure the wound size, a reference object with a known area must be included in the image; otherwise, the photo-taking distance must be restricted to a fixed range. Without a given reference, capturing the wound from a distance that is too far or too close can lead to inaccurate wound size estimation. For this purpose, we apply AI to detect a QR code, which was originally printed for patient identification. The QR code’s fixed size and distinct pattern make it a reliable reference for area calculation.

In outpatient clinical practice, clinicians often encounter the challenge of assessing an abundance of patients’ wounds within a short period of time. For this reason, establishing a systematic method for wound evaluation is crucial to enhance clinical efficiency and provide consistent and high-quality care. Additionally, patient identification became another significant concern in recent clinical practice. To address this issue, we propose an implementation of a patient’s identification code, which links each patient’s wound image to their medical record identifiers. This approach improves the convenience of wound assessment, ensures precise tracking of wound conditions, and hence significantly reduces the risk of misidentification errors.

In addition to the points mentioned above, the main purpose of our study is to develop advanced computer algorithms specifically tailored to address the issue of visual inconsistencies resulting from varying shooting distances. While the textbook *Plastic Surgery* by Peter C. Neligan outlines the correct techniques for clinical photography—including proper angles, distances, camera resolution, and more—in practice, it is challenging to ensure that every photograph is captured without error. For this purpose, a convenient and reliable photo-taking technique has been introduced, which involves capturing wound images from a perpendicular angle [13]. The consistency of the camera-to-wound distance can be disregarded without compromising accuracy. Our goal is to reduce the burden on clinicians and nurses by allowing them to take wound photos quickly, without the need to repeatedly adjust the focus or camera distance.

## 2. Materials and Methods

### 2.1. Participants

This study involved patients with visible skin wounds who attended the outpatient clinic. A total of 40 patients with clearly exposed skin wounds were selected as the study sample. Inclusion criteria required the wounds to be flat, uncovered, and suitable for photographic documentation. Additionally, the patient’s identification sticker, containing a QR code, was positioned within the designated reference area during image capture.

Exclusion criteria: Patients were excluded if their wounds had excessive obstructive elements, such as extensive bandages or fluid accumulation, which could interfere with image analysis.

### 2.2. Wound Area Analysis

We used K-means clustering to analyze the wound area to understand the recovery condition and prognosis of the wound [14]. The algorithm can segment the wound region by clustering pixels in an image based on their color similarity. Since placing a patient’s identification QR code or barcode near the wound prior to photography is a routine practice in clinical settings, this study utilizes the unique pattern of the QR code as a reference point for determining the wound size. As a result, this approach does not require any additional effort from medical professionals. Since QR codes possess a unique graphical pattern, they can be easily detected in wound photographs using image-processing techniques, enabling the calculation of their total pixel area [6]. With the total pixel area of the wound, a ratio can be derived by comparing it to the actual pixel area of the QR code on the identification sticker. This ratio is then used for the calculation of the actual wound area based on the pixels within the wound’s boundaries [15].

$$Wound\;actual\;area=\frac{Wound\;pixel\;area\times QR\;code\;actual\;area}{QR\;code\;pixel\;area}$$

### 2.3. QR Code Detection Algorithm

**Figure 2:**
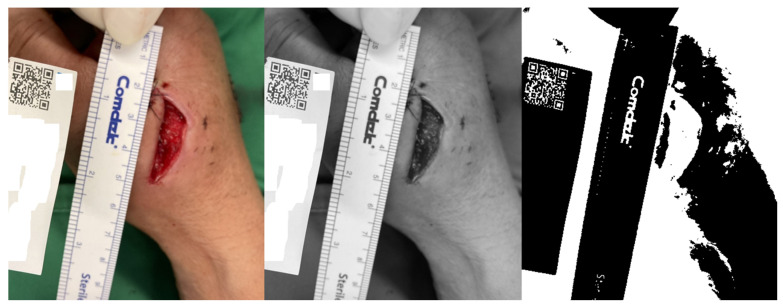
An inverted binary image with the patient’s identity removed and the distinct pattern of the QR code detected.

After uploading the photo, we convert it into a grayscale image at first. This is followed by binary inversion transformation, applied through a thresholding operation. This process creates a binary image in which each pixel is assigned a value of 1 (white) or 0 (black), depending on whether its intensity is above or below a specified threshold. To automatically select the optimal threshold value, we apply Otsu’s method—a well-known technique in computer vision and image processing for automatic image thresholding [16]. Since we are using an inverted binary image, pixels with a value of 1 are converted to 0, and pixels with a value of 0 are converted to 1. The result of this process is illustrated in Figure 2.

**Figure 3:**
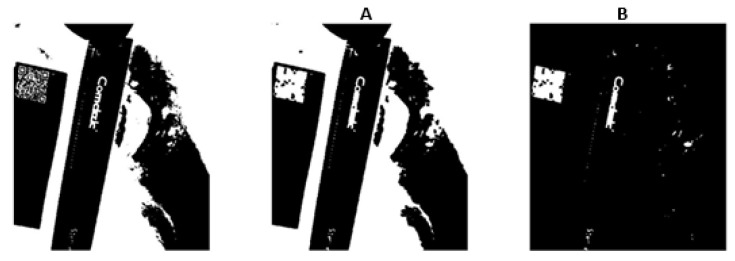
(**A**) An inverted binary image obtained after dilation and erosion. (**B**) A binary image after removing objects that are connected to the border.

Next, we use a dilation operation followed by an erosion operation in image processing, which can be used for closing small gaps, filling holes, or connecting disjoint elements in an image. The dilation expands the white regions in the binary image, while the erosion shrinks the expanded regions back to their original size, effectively closing small gaps and holes [17]. Due to the unique pattern of QR codes, this method makes the boundaries of the QR code more distinct, as shown in Figure 3A. 

Next, we will remove objects that are connected to the border of a binary image. This can be useful for cleaning up the image by removing unwanted objects or noise that touch the image’s border. The resulting image will only show the interior objects that do not touch the image borders, as shown in Figure 3B. This step helps further define the boundaries around the QR code, allowing its area to be more easily calculated through image processing in the following steps. This function can be achieved using MATLAB (R2025a) image processing.

**Figure 4:**
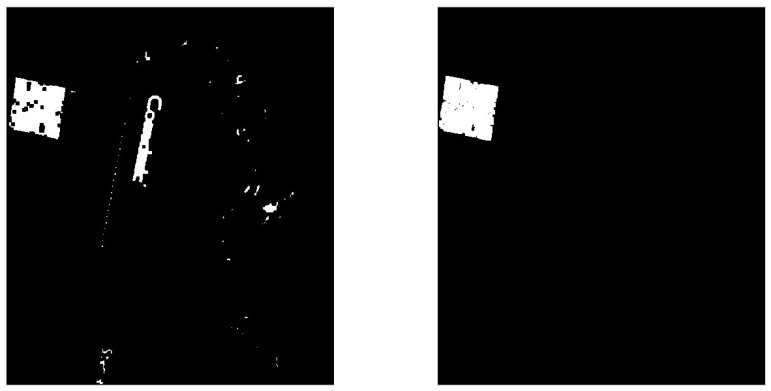
A binary image after applying an area filter to black out background noise (unwanted information).

Afterwards, we use an area filter to filter out noise or keep only objects of a certain size. By applying this function, we can retain only the objects with an area that falls within a specified range. This method will leave only the area of the QR code in the image, as shown in Figure 4, making it easier to calculate the number of pixels contained within that area. We can easily calculate the total number of pixels with a pixel value of 1 (white) to record how many pixels are contained within the area of the QR code in this image [18].

### 2.4. Wound Boundary Detection Algorithm

**Figure 5:**
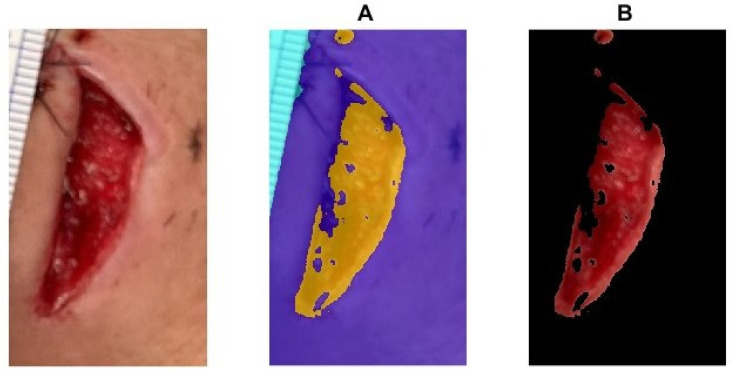
(**A**) The image is converted into the L*A*B* color space. (**B**) The boundary of the wound.

To calculate the boundary area of a segmented region in an image, the process begins by segmenting the image into meaningful regions. For example, the image is first converted into the L*A*B* color space, which separates brightness from color, improving segmentation accuracy (as shown in Figure 5A) [19,20]. K-means clustering is then applied to group the image pixels into distinct clusters, and the cluster corresponding to the wound region is identified to create a binary mask [20]. To refine this mask, morphological operations such as opening (to remove small objects) and hole filling are applied, resulting in a cleaned binary representation of the wound. Once the segmented wound region is finalized, ‘bwboundaries’ is used in MATLAB to trace the boundary points of the binary region, identifying the outline of the wound (as shown in Figure 5B). To calculate the enclosed area, we count the pixels that are not black within the wound cluster, which gives the pixel area of the segmented region. The wound area measurements can be carried out by converting the pixel area into cm^2^ using a reference scale, represented by a QR code in our study. This approach effectively combines clustering, morphological processing, and boundary detection to isolate and measure the wound’s area.

### 2.5. Verification of the Accuracy of the Proposed Algorithm

**Figure 6:**
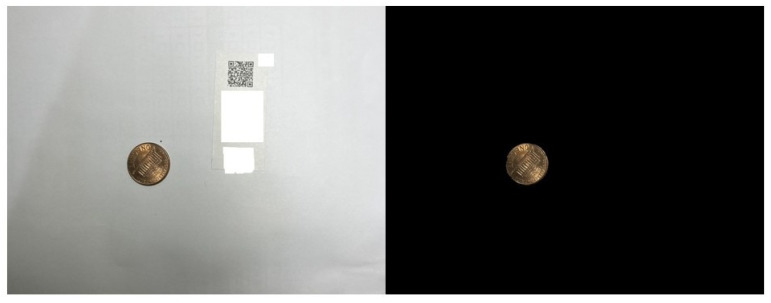
The AI detects the one-cent coin and blacks out the background.

**Figure 7:**
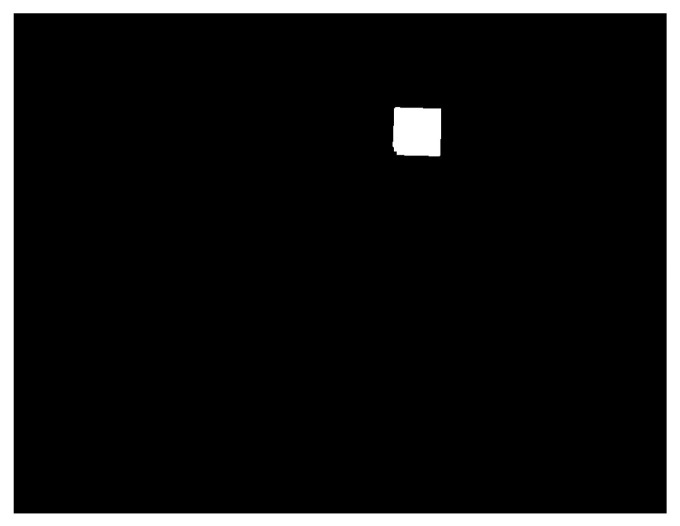
The QR code detected by AI is highlighted in white.

First, it is necessary to validate the accuracy of the proposed algorithm in determining the wound area. To validate it, a one-cent coin was used as a reference standard, shown in Figure 6. Our algorithm calculates pixel areas of a one-cent coin and a QR code, captured Figure 7, at three different random heights. We then compute the ratio by dividing the coin’s pixel area by that of the QR code and take the average of these ratios, as presented in Table 1. The resulting average ratio from the three values is 1.9699.

By multiplying this ratio by the QR code’s area (1.2 cm × 1.2 cm), we estimate the coin’s area to be 2.8366 cm^2^, which represents a 0.4% difference from the actual area of a one-cent coin (2.8488 cm^2^). Based on these findings, we consider the algorithm to be sufficiently accurate. In the next phase, we will apply this algorithm to assess the wound area across different cases. Paired *t*-tests, Pearson correlation, and ANOVA were used to verify that there were no significant differences in wound area measurements across three randomly captured images of 40 wounds.

## 3. Results

### Wound Area Assessment

**Figure 8:**
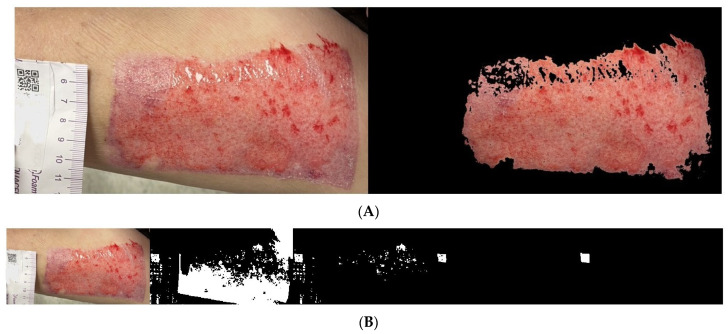
(**A**). The same wound, with the 1st photo taken at a random height. (**B**). Photos from the 1st shot: step-by-step images based on our proposed approach.

**Figure 9:**
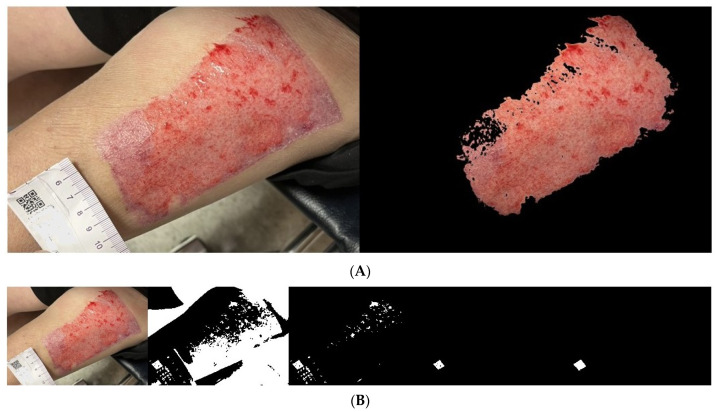
(**A**) The same wound, with the 2nd photo taken at a random height. (**B**). Photos from the 2nd shot: step-by-step images based on our proposed approach.

**Figure 10:**
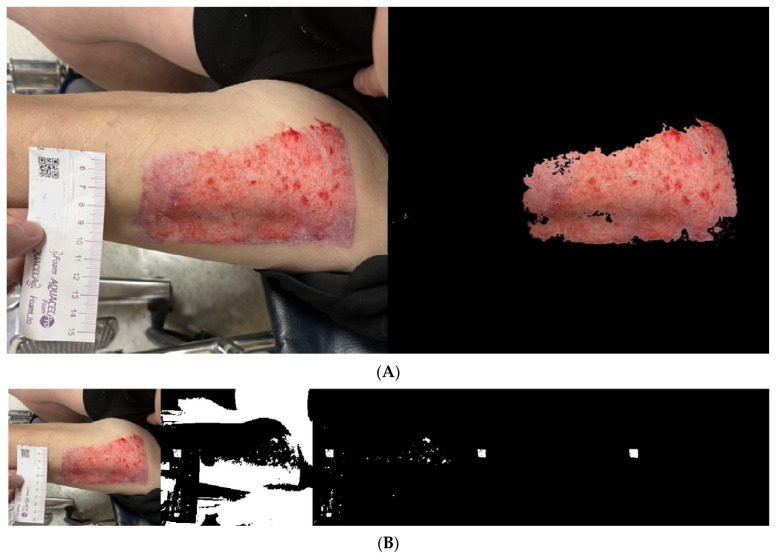
(**A**). The same wound, with the 3rd photo taken at a random height. (**B**). Photos from the 3rd shot: step-by-step images based on our proposed approach.

We used the proposed algorithm to evaluate the wound areas of 40 patients. For each patient’s wound, we randomly captured three images at different heights using an iPhone, as illustrated in Figure 8A, Figure 9A and Figure 10A with a single patient’s wound as an example. Figure 8B, Figure 9B, and Figure 10B illustrate the step-by-step process based on our proposed method. Starting with the leftmost original image taken with a smartphone, each subsequent step to the right demonstrates how the QR code area is detected and unrelated noise is filtered out. This enables a clearer and more accurate calculation of the QR code’s pixel area in the image.

**Figure 11:**
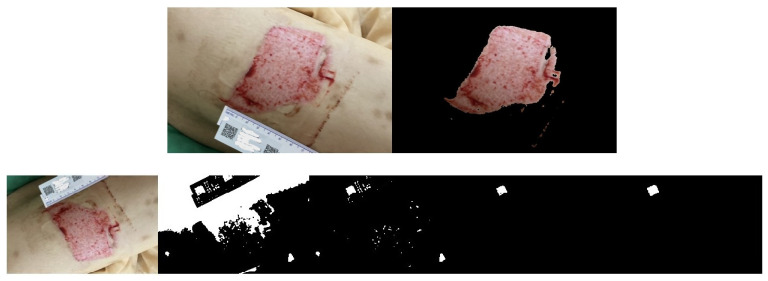
Another wound example. Photos from the 1st shot at a random height: step-by-step images based on our proposed approach.

**Figure 12:**
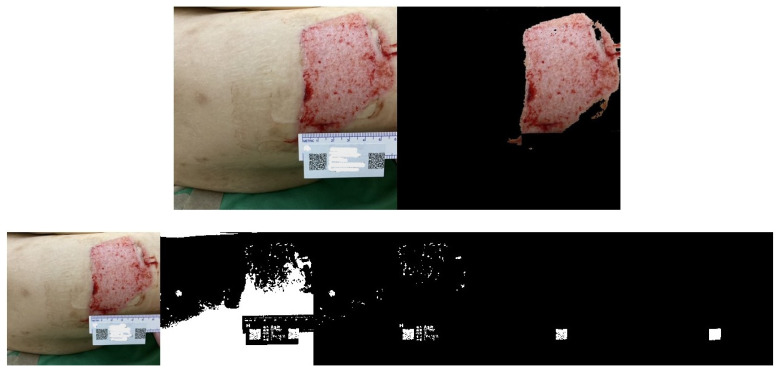
Another wound example. Photos from the 2nd shot at a random height: step-by-step images based on our proposed approach.

**Figure 13:**
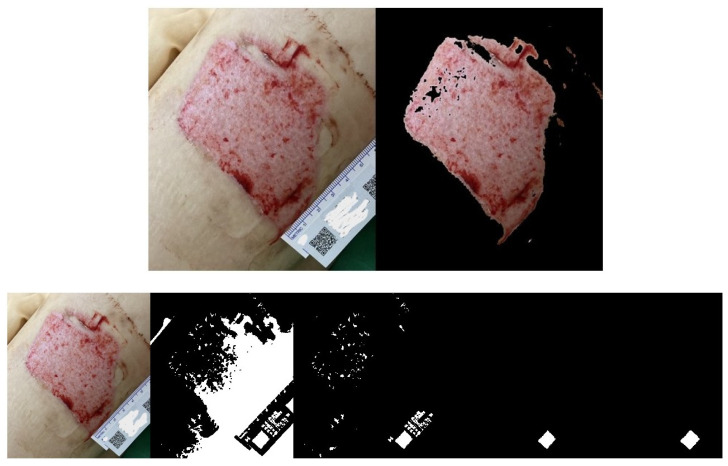
Another wound example. Photos from the 3rd shot at a random height: step-by-step images based on our proposed approach.

Another set of three randomly captured images of the other wound, taken with a different smartphone device (Asus phone), is shown in Figure 11, Figure 12 and Figure 13. Our proposed AI-assisted method can clearly outline the wound boundary and accurately detect the QR code location, even when photos are taken at different heights. It also blacks out unwanted noise, allowing us to calculate the pixel area occupied by the QR code in the image easily. By comparing this pixel area to the QR code’s actual size, we can estimate the real area of the wound.

Table 2 represents an analysis of the ratio of wound area pixels to QR code area pixels, including the mean, standard deviation, Pearson correlation, and ANOVA. The mean and standard deviation of the calculated ratios for the first, second, and third captures were 29.43 ± 5.40, 29.49 ± 5.46, and 29.55 ± 5.51, respectively. Moreover, the Pearson correlation between the first and second photos shot shows an almost perfect correlation (0.997), indicating that their results are nearly identical. Similarly, the Pearson correlations between the first and third photos shot, as well as between the second and third photos shot, are also very strong (>0.99), demonstrating high consistency across all three tests. Therefore, the three tests are strongly correlated, confirming that our proposed algorithm estimates the same wound in a highly consistent manner.

In Table 3, the paired *t*-test comparing the first and second photos shot resulted in a *t*-value of −0.908 and a *p*-value of 0.370, indicating no significant difference between the two. Similarly, the *t*-test comparing the first and third photos shot yielded a *t*-value of −1.367 and a *p*-value of 0.179, also showing no significant difference. The *t*-test for the second and third photos shot resulted in a *t*-value of −0.608 and a *p*-value of 0.547, indicating no significant difference between these as well. Since all *p*-values are greater than 0.05, none of the test pairs show statistically significant differences. This suggests that the three tests produce similar results and can be considered equivalent. Lastly, we conducted an ANOVA test to determine whether there were significant differences among the three tests. The F-statistic was 0.0049, indicating minimal variation between groups. The *p*-value was 0.9951, which is much greater than 0.05, confirming that there is no significant difference among the three tests. Based on the results, we conclude that our algorithms are highly reliable, consistent, and produce accurate results regardless of the height at which the photos are taken.

## 4. Discussion

Currently, a variety of methods are available for assessing the wound size, each with its own advantages and limitations depending on the clinical setting and wound characteristics [21]. In cases of extensive burn injuries, appropriate fluid therapy and protein supplementation were found to be essential for effective management and recovery [22]. Furthermore, accurate wound size measurement is essential for selecting the appropriate flap in wound reconstruction procedures.

Based on our findings, wound assessment can be performed in a more precise and efficient manner. Our results reinforce the understanding that the wound size is a critical factor in shaping treatment strategies and has a significant impact on the patient prognosis [23,24]. Continuous wound monitoring is a critical aspect of care, particularly in patients undergoing flap reconstruction [25].

Before conducting this research, it was essential to verify the accuracy and reliability of our proposed algorithm. To achieve this, we selected U.S. coins with standardized dimensions for verification. After performing the necessary calculations, we confirmed the precision of our system. The second challenge involved addressing variations in images captured by different photographers. Traditional guidelines emphasize the need for consistency in the shooting distance, camera specifications, and angles. Typically, patients must be positioned in a designated photography area and stand at a specific location to ensure uniformity in image capture. However, in a busy clinical environment, it is challenging to enforce strict adherence to these photography guidelines for every patient.

To address this, our research design focused on finding a way to capture images quickly and accurately while minimizing human-induced errors in the shooting process. We developed a method in which a patient identification barcode of a fixed size is placed around the wound as a reference for area measurement. This approach allows the photographer to concentrate solely on maintaining a fixed angle (perpendicular to the wound), ensuring that variations in the shooting distance do not compromise the accuracy of wound size measurements. With the use of patient identification through a QR code or barcode system, its integration into our algorithm does not add any additional burden to the clinical workflow. By integrating this with photography-based wound assessment, medical staff can evaluate the wound size without the need for extra reference markers or manual tracing with transparent sheets. The process requires only a camera and the patient’s identification code from their medical records, making it a simple and efficient solution.

Since our primary goal is to determine the wound size, it is not necessary to use the same camera for capturing images. Although different smartphones may have varying camera resolutions and image quality, as long as the captured photo includes both the wound and the patient’s QR code (used as a reference), the wound area can still be accurately analyzed. The measurement of the wound area is not affected by the camera’s pixel quality.

Therefore, when the patient needs a follow-up on the wound size in the future, clinicians or nurses can use any available smartphone to capture a photo and analyze the current wound area.

This approach enables different operators to easily and quickly capture images using their own mobile devices. With the increasing interest in computer-assisted wound assessment systems, our study is the first to utilize patient identification codes as a reference standard for wound evaluation. We hope that our research design and methodology can serve as a valuable reference for future healthcare professionals and system developers.

### 4.1. Limitations

Despite promising initial results, our study faced several challenges that warrant attention.

First, the system currently lacks the capability to assess the wound depth, which limits its utility in evaluating wounds requiring volumetric measurements.

Second, image acquisition is sensitive to the angle of capture. To maintain measurement accuracy, we instructed photographers to take images perpendicularly to the wound surface and avoid angled shots. Deviations from this guideline can lead to perspective distortion, affecting both size estimation and segmentation performance [26,27].

Third, wound care is a highly specialized domain that encompasses complex physiological factors, such as blood circulation, oxygenation levels, moisture balance, and signs of infection. Our system is currently unable to assess these critical indicators, which typically require the expertise of experienced clinicians [28].

In terms of dataset size, our study includes wound images from 40 patients. Although limited, this sample size is appropriate for a preliminary study, whose goals are to identify patterns, assess feasibility, and refine the methodology prior to scaling up the research.

Another significant limitation lies in the use of K-means clustering for wound boundary detection. K-means struggles in low-contrast scenarios where the color or intensity difference between the wound and surrounding tissue is minimal, often leading to inaccurate segmentation and boundary misclassification [29]. This issue is exacerbated under poor lighting conditions, which further degrade contrast and hinder performance.

Moreover, K-means clustering operates solely on color and intensity features, ignoring critical information such as depth and texture. Given that wounds often have irregular surfaces and heterogeneous tissue composition, the absence of depth and texture analysis diminishes the accuracy of wound segmentation [30].

Overall, while our system shows potential, addressing these limitations will be essential to improving its clinical relevance and scalability.

### 4.2. Recommendations

To improve wound visualization, consistent and adequate lighting during image capture is essential. A well-lit environment minimizes shadows and uneven illumination, both of which can negatively impact segmentation accuracy. The use of camera flash is discouraged, as it may cause glare and obscure wound details. Instead, controlled clinical lighting or a ring light is recommended to enhance image quality and ensure more reliable wound assessment [31,32,33].

In addition to proper lighting, minimizing background distractions is vital. A neutral, non-reflective surface surrounding the wound area helps reduce segmentation errors. If needed, a standardized imaging template or drape can be employed to isolate the wound from its surroundings, further supporting accurate image analysis.

Looking ahead, we plan to incrementally expand our dataset by including a broader range of wound types and healing conditions. This expansion will strengthen the robustness of our findings and improve the generalizability of our approach, ultimately advancing its readiness for clinical integration.

As telemedicine becomes increasingly integral to modern healthcare, there is a growing demand for remote wound assessment tools, driven by the rising prevalence of chronic wounds—largely associated with aging populations, diabetes, and obesity [34]. AI has demonstrated strong potential in improving the accuracy and efficiency of wound assessments, particularly by enabling better differentiation between chronic wounds and other skin conditions. This capability supports more personalized and effective treatment plans.

It also allows patients to take photos of their wounds to evaluate the healing progress based on the wound area. These images can be uploaded to the hospital system, enabling physicians to remotely monitor the wound’s condition. This feature offers significant convenience in today’s era of IoT-based medical applications.

From a healthcare system perspective, integrating AI can help reduce costs by streamlining workflows and decreasing the need for invasive procedures. It also enables real-time monitoring of wound healing, allowing for timely therapy adjustments and early detection of complications—ultimately improving clinical outcomes and patient safety.

Our QR code-based wound measurement method holds potential for adaptation into a mobile application, allowing patients to capture wound images at home and securely transmit them to clinicians for remote evaluation [35]. This model could significantly benefit patients with chronic wounds—such as diabetic foot ulcers and pressure ulcers—for whom frequent in-person visits may be challenging.

Future work could explore the development of an AI-powered mobile system capable of delivering real-time wound size estimation, monitoring healing progress, and issuing alerts in cases of wound deterioration [36]. Such advancements would mark a step forward in empowering both patients and healthcare providers with accessible, tech-enabled wound care solutions.

## 5. Conclusions

This study presents a novel and objective method for wound area assessment using K-means clustering and a QR code as a reference, which offers several advantages over traditional wound measurement techniques. By leveraging image processing, this approach eliminates subjectivity, ensures reproducibility, and enhances accuracy in wound area quantification. The results of our validation tests indicate that this method provides highly consistent wound size measurements, independent of variations in photo-taking distances and different medical practitioners. Our findings demonstrate that the combination of K-means clustering and QR code referencing is a reliable, efficient, and scalable solution for wound area measurement in outpatient settings. This technology has the potential to significantly improve the clinical workflow, enhance patient outcomes, and provide a foundation for future advancements in automated wound assessment systems.

## Figures and Tables

**Table 1:** Coin area and QR code pixel count.

Photo Shooting	Coin Area Pixel	QR Code Pixel	Ratio
1st	10,806	5470	1.9755
2nd	13,831	6958	1.9877
3rd	12,050	6190	1.9466

**Table 2:** The mean, standard deviation, Pearson correlation, and ANOVA results from the analysis of the ratio of wound area pixels to QR code area pixels.

	Mean ± Std	Pearson Correlation	ANOVA F-Statistic	ANOVA *p*-Value
1st photo shot	29.43 ± 5.40	-	0.0049	0.9951
2nd photo shot	29.49 ± 5.46	0.997 (vs. Test 1)		
3rd photo shot	29.55 ± 5.51	0.995 (vs. Test 1), 0.993 (vs. Test 2)		

**Table 3:** The paired *t*-tests comparing the first vs. second, first vs. third, and second vs. third photos shot.

	*t*-Statistic	*p*-Value	Significant Difference? (*p* < 0.05)
1st photo shot vs. 2nd photo shot	−0.908	0.370	No
1st photo shot vs. 3rd photo shot	−1.367	0.179	No
2nd photo shot vs. 3rd photo shot	−0.608	0.547	No

## Data Availability

The original contributions presented in this study are included in the article. Further inquiries can be directed to the corresponding author.
